# Processing Characteristics of Micro Electrical Discharge Machining for Surface Modification of TiNi Shape Memory Alloys Using a TiC Powder Dielectric

**DOI:** 10.3390/mi11111018

**Published:** 2020-11-20

**Authors:** Ziliang Zhu, Dengji Guo, Jiao Xu, Jianjun Lin, Jianguo Lei, Bin Xu, Xiaoyu Wu, Xujin Wang

**Affiliations:** 1 Institute of Semiconductor Manufacturing Research, Shenzhen University, Nan-hai Ave 3688, Shenzhen 518060, China; ziliang_zhu@163.com (Z.Z.); im.jianjun@szu.edu.cn (J.L.); wanghoshi@szu.edu.cn (X.W.); 2 Guangdong Provincial Key Laboratory of Micro/Nano Optomechatronics Engineering, College of Mechatronics and Control Engineering, Shenzhen University, Nan-hai Ave 3688, Shenzhen 518060, China; ljg_sc111@163.com (J.L.); binxu@szu.edu.cn (B.X.); wuxy@szu.edu.cn (X.W.)

**Keywords:** micro-EDM, TiNi shape memory alloy, TiC powder, surface modification, microhardness

## Abstract

Titanium-nickel shape memory alloy (SMA) has good biomedical application value as an implant. Alloy corrosion will promote the release of toxic nickel ions and cause allergies and poisoning of cells and tissues. With this background, surface modification of TiNi SMAs using TiC-powder-assisted micro-electrical discharge machining (EDM) was proposed. This aims to explore the effect of the electrical discharge machining (EDM) parameters and TiC powder concentration on the machining properties and surface characteristics of the TiNi SMA. It was found that the material removal rate (MRR), surface roughness, and thickness of the recast layer increased with an increase in the discharge energy. TiC powder’s addition had a positive effect on increasing the electro-discharge frequency and MRR, reducing the surface roughness, and the maximum MRR and the minimum surface roughness occurred at a mixed powder concentration of 5 g/L. Moreover, the recast layer had good adhesion and high hardness due to metallurgical bonding. XRD analysis found that the machined surface contains CuO_2_, TiO_2_, and TiC phases, contributing to an increase in the surface microhardness from 258.5 to 438.7 HV, which could be beneficial for wear resistance in biomedical orthodontic applications.

## 1. Introduction

TiNi SMAs have broad application prospects in the aerospace, biomedical, and automobile fields due to their excellent biocompatibility, superelasticity, shape memory effect, and wear resistance [1]. Because the Young’s modulus of titanium-nickel alloys is lower than that of other biomedical implant materials, they are widely used for medical implants [2]. In clinical medical applications, product safety and reliability are the primary requirements for long-term implants. However, amino acids and proteins in bodily fluids will accelerate metal corrosion, promoting the release of toxic nickel ions [3]. The release of metallic ions is detrimental to osseointegration and ultimately causes clinical failure [4]. Therefore, the surface modification of titanium-nickel alloys plays an important role in improving corrosion resistance and surface biocompatibility.

Previous studies have shown that a thin surface layer of titanium oxide (2–20 nm) will naturally form on the surface of TiNi alloys, and this layer can act as a barrier to human body corrosion and chemical reactions to limit the diffusion of nickel ions [5]. However, this film is unstable in the human body’s complicated and volatile environment and can easily corrode and fall off the alloy material. Therefore, surface treatment techniques have been developed to treat TiNi alloys. Titanium oxide film has good blood compatibility [6] and is biologically inert [7], and it can effectively prevent the precipitation of nickel ions. Several surface treatment methods have been used commercially, such as anodic oxidation [8], plasma immersion ion implantation (PIII) [9], coating [10], and electrochemical polishing [11]. Qin et al. [12] used a glycerol electrolyte to obtain TiO_2_ nanotubes on the surface of the TiNi alloy through anodic oxidation, which effectively improved the biocompatibility of the alloy.

Electrical discharge machining (EDM) is an unconventional machining technology that uses a series of pulse discharges between the tool and workpiece to process the workpiece [13]. It is mainly used for high-precision processing of difficult-to-cut materials. Wyszynski et al. [14] realized the high-precision micro-hole machining of cubic boron nitride and determined the optimal parameters. Wu et al. [15] developed a cut-side micro-tool suitable for the micro-EDM system and successfully realized the deep and high aspect ratio micro-holes machining on tungsten cemented carbide. In order to study the machining mechanism of micro-EDM, Liu et al. [16] analyzed the polarity effect of micro-EDM based on the movement characteristics of electrons and positive ions in the discharge plasma channel. Almacinha et al. [17] established an electro-thermal model for a single discharge of an electric discharge machining process based on the Joule heating effect theory. Roy et al. [18] analyzed the physical phenomenon behind occurrences of unusually high discharging points in reverse micro EDM by establishing a numerical model of ions and electrons’ movement in the dielectric during machining. To realize the micro-EDM machining of the three-dimensional structure, Roy et al. [19] used reverse micro EDM to generate different shapes of protruded micro features, such as 3D hemispherical and 3D coni-spherical shapes. To study EDM’s surface characteristics, Hsieh et al. [2] showed that the EDM process could successfully machine the ternary TiNiZr SMAs while ensuring its shape recovery ability. The recast layer generated on the machined surface can adhere to the substrate effectively by surface alloying, enhancing wear resistance [5]. Peng et al. [3] have reported that EDM can form a nanoporous biocompatible layer on the surface of Ti-6Al-4V, which is conducive to cell growth and proliferation.

To develop improved surface modification technologies, a new method of TiC-powder-assisted micro-EDM is proposed for the formation of a titanium oxide surface, and experiments were performed on a TiNi SMA. The effects of PMEDM parameters on the machining characteristics of TiNi SMA were investigated experimentally, and then, the surface roughness, surface morphology, and microhardness were characterized by relevant characterization techniques. Finally, the thickness and composition of the recast layer were studied deeply.

## 2. Principle and Mechanisms

**Figure 1:**
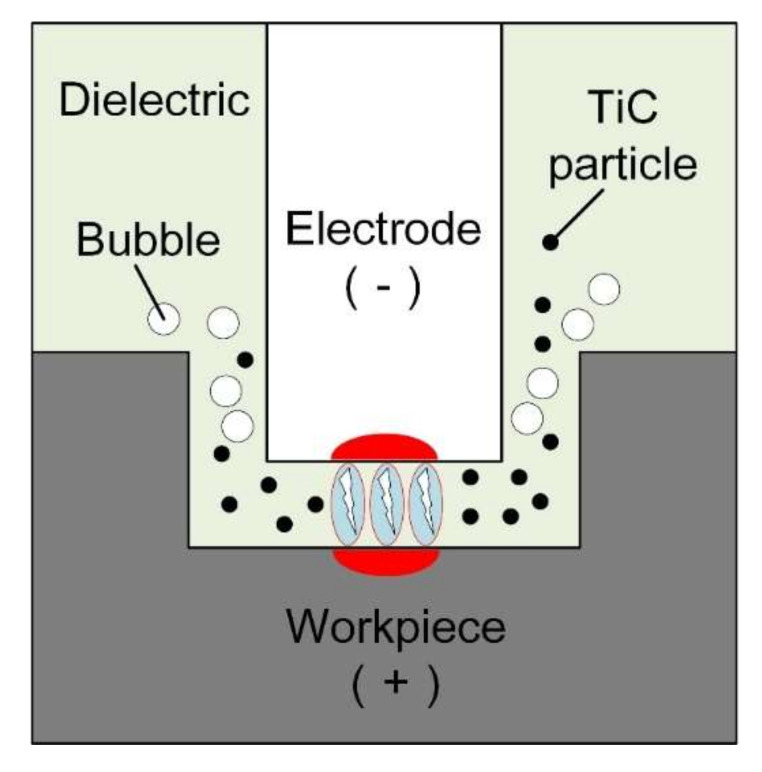
Principle of powder mixed EDM.

**Figure 2:**
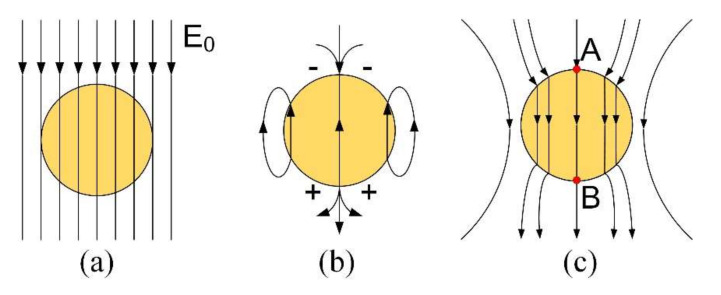
Illustration of electric fields: (**a**) uniform electric field, (**b**) electric field of the bound charge, (**c**) superimposed electric field.

Figure 1 illustrates the principle of the EDM process with TiC powder. By adding TiC-mixed powder particles with a particle size of 2 µm in deionized water, the tool electrode utilizes reciprocating movement to complete the powder-mixed EDM (PMEDM). To study the mechanism of the PMEDM, the following two assumptions were made: (1) the TiC-mixed powder particles are spherical, and (2) the electric field is an electrostatic field, shown as yellow circles and black lines in Figure 2a, respectively.

According to the principle of electronics [20], the conductive particles are polarized into bound charges under an electric field’s action. When high voltage is applied between the electrode and workpiece, the TiC particles are polarized under the action of the electric field and become a bound charge. According to the electrostatic field theory [21], no matter how strong the electric field is applied to the conductor, the electrostatic balance of the conductor will make the internal field strength of the conductor zero. In order to achieve electrostatic equilibrium, the inside of the TiC particle will generate an electric field opposite to the uniform electric field, E0, as shown in Figure 2b. Therefore, the superposition of the electric field generated by the bound charge and the external electric field distorts the uniform electric field between the electrode and workpiece, as shown in Figure 2c. When the electric field’s direction generated by the bound charge overlaps with the direction of the uniform electric field, the actual electric field intensity will reach the maximum value, i.e., points A and B in Figure 2c, and discharge breakdown takes priority here. The maximum value is given by the following [22]:

(1) $$E_{max}=\left[1+2\left(\frac{\varepsilon_{2}-\varepsilon_{1}}{\varepsilon_{2}+2\varepsilon_{1}}\right)\right]E_{0},$$

where $\;\varepsilon_{1}$ is the dielectric coefficient of the dielectric fluid, $\;\varepsilon_{2}$ is the powder’s dielectric coefficient, and E0 is the electric field strength of the uniform electric field.

The electrostatic field theory shows that the electric field intensity inside an ideal conductor is zero, and its relative dielectric constant is infinite [21]. Therefore the dielectric coefficient, $\;\varepsilon_{2}$, of the conductor tends toward infinity, and takes the limit based on Equation (1) to reach the maximum value, Emax, as follows:

(2) $$E_{max}\approx 3E_{0}.$$

Therefore, the addition of the mixed powder increases the electric field strength between the electrode and the workpiece by a factor of three and expands the discharge gap by a factor of three, which promotes the removal of processing debris.

## 3. Experimental Methods

### 3.1. Experimental Procedure

**Figure 3:**
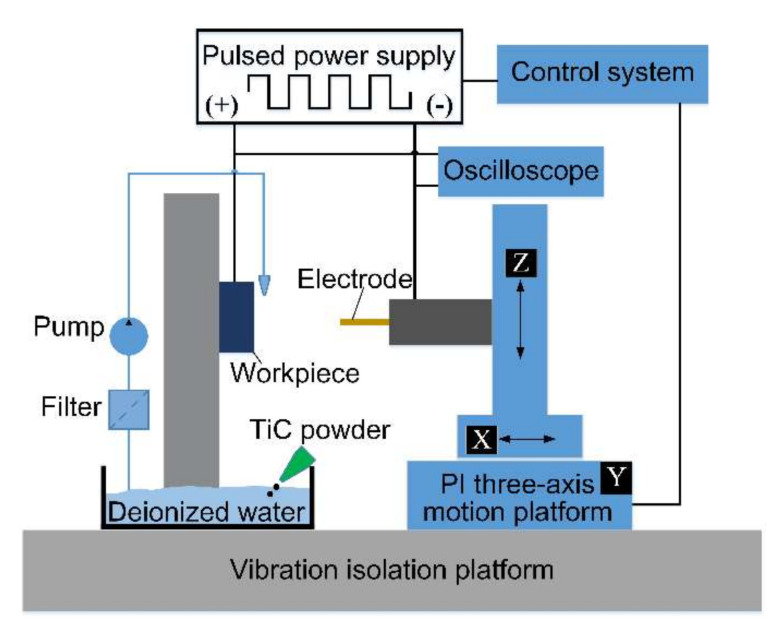
Experimental setup of powder-mixed EDM (PMEDM)**.**

Figure 3 illustrates a schematic diagram of the PMEDM equipment. During the experiment, the power supply unit included a high-voltage amplifier (Aigtek ATA-2021H, Aigtek, Xi’an, China) and a transistor-type pulse generator (Tektronix AFG3000C, Tektronix, Inc., Beaverton, OR, USA). The pulse waveform is generated by the waveform generator and amplified by a high-voltage amplifier to display the signal on the oscilloscope. The microelectrode movement in micro-EDM was realized by the three-axis micro-nano-motion platform (PI, Germany; M511.DD). A digital oscilloscope (Tektronix MDO 3000) was used to monitor the pulse signal in real-time during the machining process.

As demonstrated by Lin et al. [23], negative polarity processing can provide a larger MRR, while positive polarity processing can provide a thicker recast layer on the processed surface. Therefore, we chose to employ positive polarity processing in this study. A micropump was used to circulate and mix the dielectric fluid to ensure that the TiC powder was uniformly dispersed. The detailed experimental processing parameters are shown in Table 1.

### 3.2. Experimental Materials and Measurements

**Figure 4:**
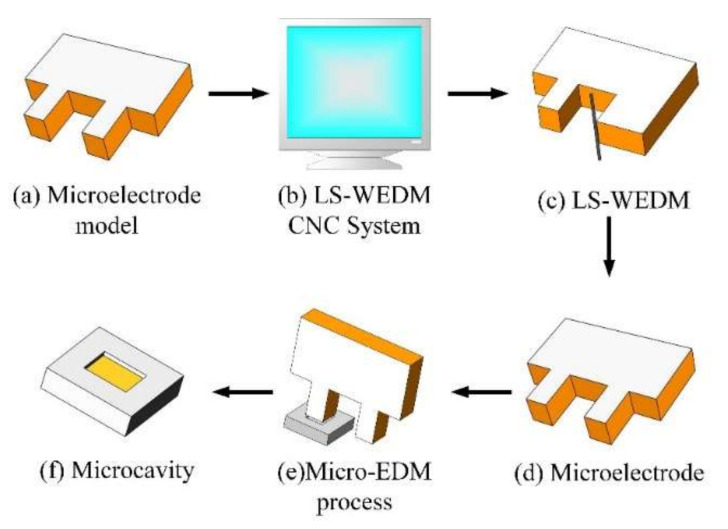
Fabrication process of microelectrode and microcavity.

In the EDM process, the workpiece material used in the experiments was TiNi SMA (China Tai’zhou Cinoo Mental material Co., Ltd.). The as-received samples were a rectangular parallelepiped with a length of 100 mm, a width of 300 mm, and a thickness of 0.5 mm. Its elemental composition and main thermophysical properties are presented in Table 2 and Table 3, respectively. Brass sheets of 1 mm thick were used as raw material for fabricating the microelectrodes. At present, deionized water is widely used as the dielectric fluid due to its weak electrical conductivity, which can also avoid carbon deposition in the spark oil. TiC powder was added to deionized water at different concentrations. Its physical properties are listed in Table 4. The fabrication of microelectrode and microcavity was shown in Figure 4, and the detailed process was as follows. First, according to the microcavity to be machined, a corresponding microelectrode model was designed (Figure 4a). Secondly, import model parameters into the low-speed wire EDM machine (LS-WEDM, Sodick, Japan; AQ250Ls) CNC System (Figure 4b) and start cutting (Figure 4c) to fabricate a single microelectrode with a size of 0.8 mm × 1 mm (Figure 4d). The microelectrode was then employed in the micro-EDM to process a microcavity with a depth of 100 µm (Figure 4e). Finally, a microcavity with a high-quality surface was obtained successfully (Figure 4f).

A scanning electron microscope (SEM) manufactured by TESCAN, Czech Republic (model: LYRA3 XMH) was used to observe the surface morphology. A laser scanning confocal microscope (LSCM, Keyence, Japan; VK-X260K) was used to measure the surface roughness. Analyses of the EDM-treated surfaces were performed at room temperature using X-ray diffraction (XRD, MiniFlex600, Rigaku Corporation, Tokyo, Japan) at a 2θ scanning rate of 3° min −1. A microVickers hardness tester (MHV-1000A, HuaXing, Lai’zhou, China) was used to measure the surface hardness under a load of 100 g for 10 s. The average hardness value was taken from at least four test readings for each specimen.

## 4. Results and Discussion

### 4.1. Discharge Waveforms Comparison of EDM and PMEDM

**Figure 5:**
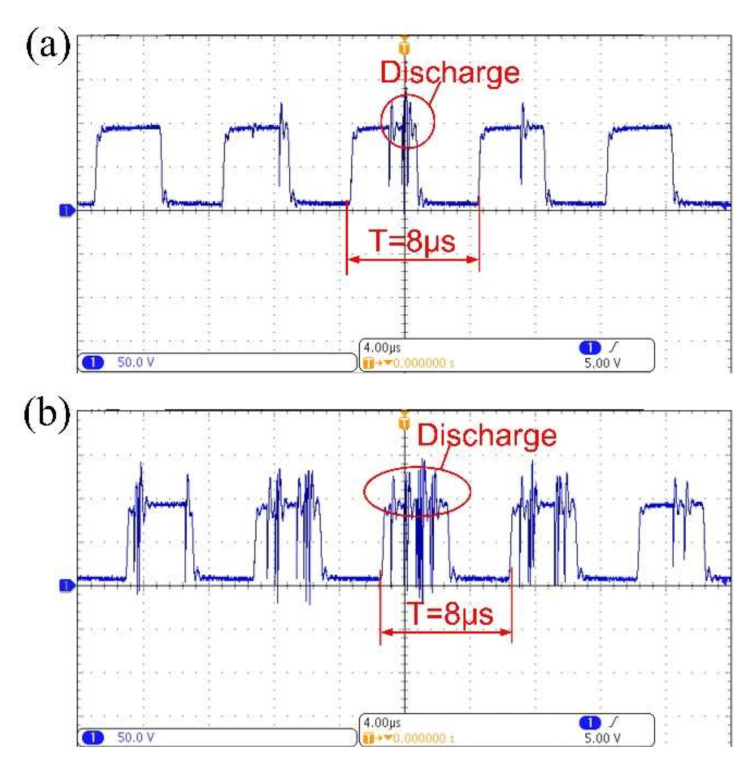
Discharge voltage waveform comparison: (**a**) without and (**b**) with the addition of TiC powder (5 g/L).

A digital oscilloscope acquired the discharge voltage waveforms to study the effect of the addition of TiC powder on the electro-discharge behavior of the material. Processing parameters, including a pulse width of 4 µs, a duty of 50%, and a machining voltage of 80 V, were determined before micro-EDM processing. The voltage waveforms for the micro-EDM were acquired by an oscilloscope for both without TiC powder addition and with the addition of 5 g/L TiC powder, as shown in Figure 5. Taking the same time (8 µs) for comparison, the number of pulses for the discharge voltage in Figure 5b was significantly higher than in Figure 5a. This result indicates that the addition of TiC powder significantly improved the discharge characteristics of the TiNi SMA in micro-EDM. Moreover, a multiple discharging effect was observed within a single period in Figure 5b, which indicates that the TiC powder refined the discharging energy.

### 4.2. Influence of Machining Process Parameters on the Material Removal Rate

The MRR was calculated as the ratio of the volume of material removed from the workpiece to the processing time (mm 3/min). The volume of material removed was obtained by analyzing the three-dimensional surface topography scanned by LSCM.

**Figure 6:**
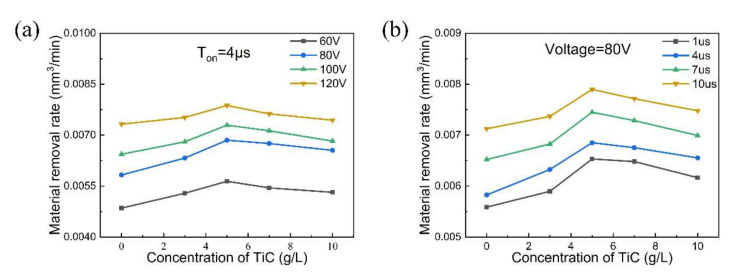
Material removal rates (MRRs) obtained with (**a**) different voltages and (**b**) different pulse durations in the TiC dielectric with concentrations of 0, 3, 5, 7, and 10 g/L.

Figure 6 shows the effect of the concentration of TiC powder on the MRR under different machining voltages and pulse widths. It is clear that the MRR increases with increasing concentration of TiC powder; regardless of the machining voltages and pulse widths, the maximum MRR is obtained at a concentration of 5 g/L. This result confirms that the discharge frequency is increased, and the discharge energy is improved by the addition of TiC powder to the dielectric fluid. In addition, when the concentration of TiC powder is greater than 5 g/L, the MRR tends to decrease. This trend agrees with that reported by Jahan et al. [24]. When the powder concentration is excessively high, the large number of conductive particles between the two poles cannot be removed easily and cause secondary sparking. Eventually, this leads to instability of the machining process and increases the machining time.

It is noted that the MMR increases with increasing machining voltage, as shown in Figure 6a. A high machining voltage can effectively increase the discharge channel’s current density, which facilitates the melting and evaporation of materials. Figure 6b also shows an increase in the MRR with the pulse duration. The pulse duration determines the level of discharge energy, and high pulse durations can provide the necessary time to transmit discharge energy. Hence, a high MRR occurs at higher machining voltages and longer pulse widths in the micro-EDM process.

### 4.3. Influence of Machining Process Parameters on the Surface Roughness

**Figure 7:**
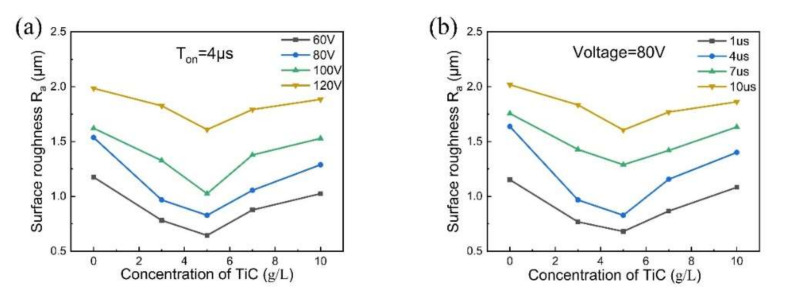
Surface roughness obtained with (**a**) different voltages and (**b**) different pulse durations in the TiC dielectric with TiC concentrations of 0, 3, 5, 7, and 10 g/L.

Figure 7 shows the effect of the concentration of TiC powder on the surface roughness under different machining voltages and pulse durations. In both cases, the surface roughness decreases with increasing TiC powder concentration up to 5 g/L; then, as the TiC powder concentration increases further, the surface roughness tends to increase. Liew et al. [25] recently showed that adding an appropriate amount of conductive powder to the dielectric fluid can uniformly disperse the discharge energy and reduce the craters’ size, thereby improving the surface finish. Nevertheless, high-concentration TiC powder tends to accumulate on the workpiece’s surface, which severely inhibits the transfer of discharge energy. Moreover, the deposited powder and melting material cannot be removed from the machining gap in time, which causes more frequent secondary sparking and circuiting. This effect will increase surface roughness.

It is noted that the surface roughness increases with increasing voltage in Figure 7a and pulse width in Figure 7b. Under low machining voltages and short pulse widths, the pits on the machined surface were small and shallow, and the melting material was easily removed. When the machining voltage and pulse width were increased, the discharge time became longer, and the single pulse discharge energy increased. This caused the radius and depth of the discharge marks to increase, leading to an increase in the surface roughness.

### 4.4. Surface Morphology of the EDM-Treated TiNi SMA

**Figure 8:**
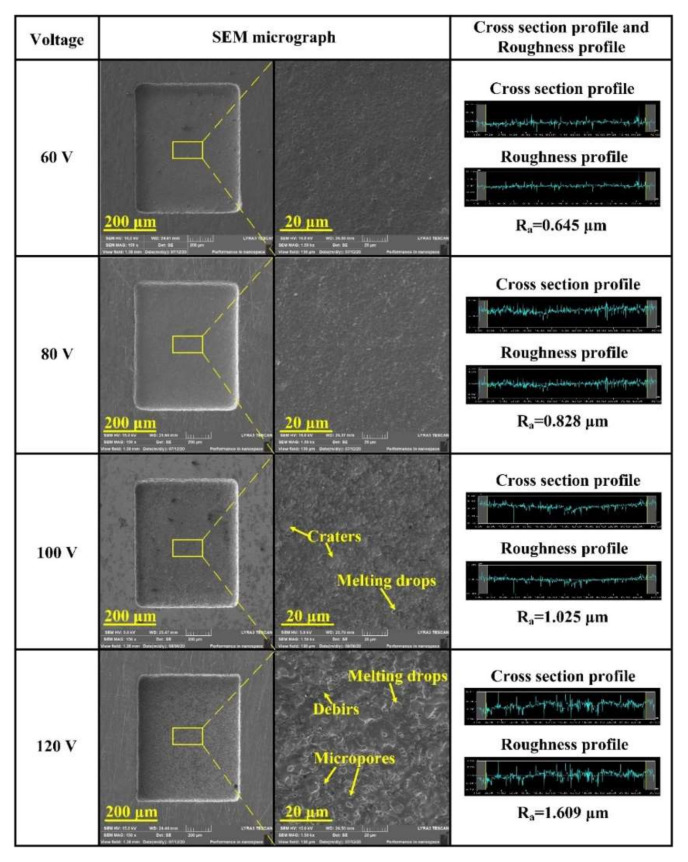
Effect of different machining voltages on surface roughness and scanning electron microscope (SEM) micrographs of the microcavities bottom.

In this study, the surface microtopography and surface roughness (Ra) was used to evaluate surface quality deviations. Five surface roughness measurements (Ra) made at different positions on the bottom of identical microcavity were averaged. Figure 8 shows SEM micrographs and surface roughness of the microcavities bottom variation with the machined voltage (60–20 V) increased under an applied pulse width of 4 µs and a TiC powder concentration of 5 g/L. When the machined voltage was 60 V, the machined surface was relatively smooth due to the low discharge energy, and the surface roughness was 0.645 µm. However, as the machined voltage increased, the surface quality gradually decreased. Until the machined voltage reaches 100 V, numerous discharge craters and melting drops were observed on the machined surface. Nevertheless, if the machined voltage increased to 120 V continuously, the machined surface became rougher, and the surface roughness up to 1.609 µm. This trend was consistent with that reported by Xu et al. [26]; the surface roughness (Ra) increases with greater machined voltage. This was because the electron flow in the channel had an enhanced bombardment effect on the anode under the condition of high voltage, and many melting drops, debris, micropores were observed on the surface.

**Figure 9:**
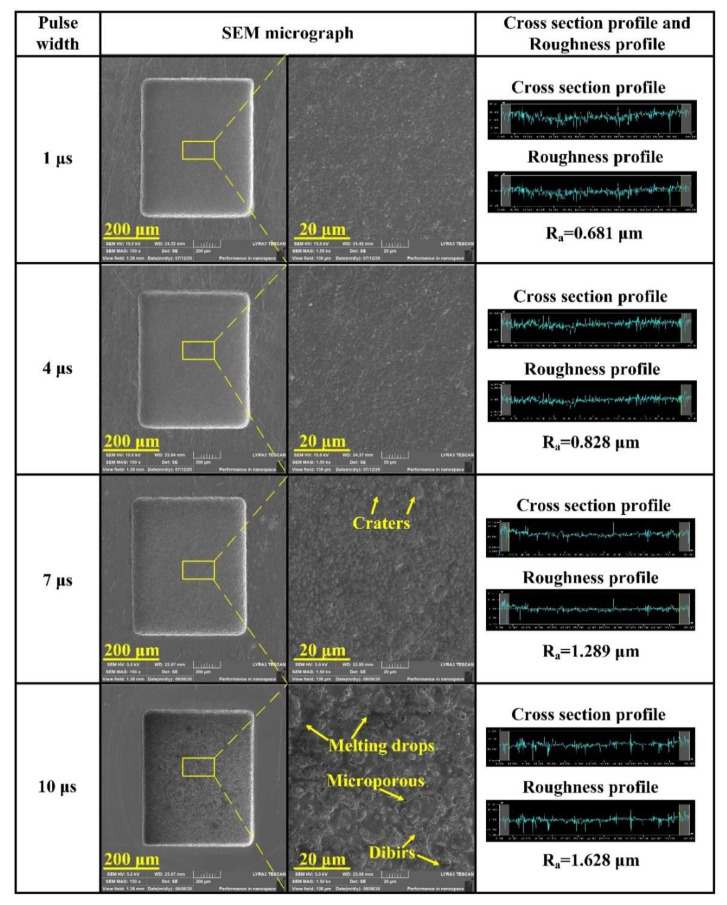
Effect of different pulse widths on surface roughness and SEM micrographs of the microcavities bottom.

Using a TiC powder concentration of 5 g/L, a machined voltage of 80 V, the influence of different pulse widths on SEM micrographs and bottom surface roughness of the microcavities were discussed in Figure 9. Similar to machined voltage, it can be found that the machined surface of a short pulse width contains shallower and smaller discharge craters compared to long pulse width; because lower pulse width has a smaller MRR, the TiC powder has enough time to refine the discharge energy at the same machining depth, resulting in a smooth bottom surface. As the pulse width increased to 10 µs, the surface roughness increased up to 1.628 µm. The analysis found that with the substantial increase of the pulse width, the current density in the discharge channel continued to increase, and the bombardment effect of charged particles was enhanced, which led to an increase in the radius and depth of the discharge marks. Moreover, the increase of discharge debris particles in long pulse width machining caused short circuits and arcing phenomenon and led to an unstable machining process [27]. Meanwhile, the melting material cools and solidifies on the surface of the workpiece during the deionization stage.

**Figure 10:**
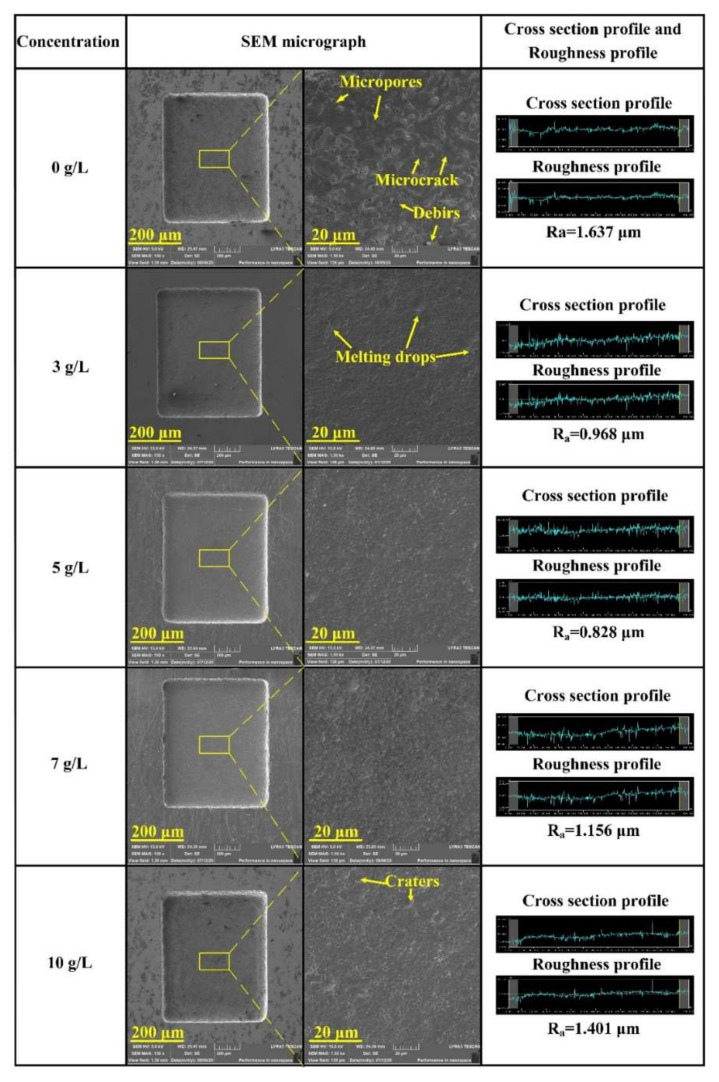
Effect of different TiC concentrations on surface roughness and SEM micrographs of the microcavities bottom.

The effect of TiC concentrations on SEM micrographs and surface roughness of the microcavities bottom under an applied machining voltage of 80 V and pulse width of 4 µs is given in Figure 10. Results indicate that the addition of TiC powder improved the surface quality, and the surface roughness was always lower than that of the material without the addition of mixed powder. When the TiC concentration was approximately 5 g/L, the minimum surface roughness was Ra 0.828 µm. According to Bui et al. [28], the addition of powder reduces the dielectric resistivity and increases the discharge gap. Conductive particles such as graphite [29], cobalt [30], and molybdenum [31] can form chains across the electrodes and enlarge the gap distance, which not only allows more working fluid to flow through but also lowers the single pulse explosion pressure, resulting in smaller and shallower craters. As mentioned in Section 4.1, TiC powder can disperse the discharge energy and increase the discharge gap. The discharge distribution becomes more uniform. Furthermore, many debris, micropores, and microcracks were observed on the machined surfaces with the pure dielectric fluid. According to the measurement results of surface roughness, when using higher TiC concentrations, such as 10 g/L, the sizes of the discharge craters are increased compared with those obtained with lower TiC concentrations.

### 4.5. Influence of Micro-EDM Parameters on the Recast Layer

**Figure 11:**
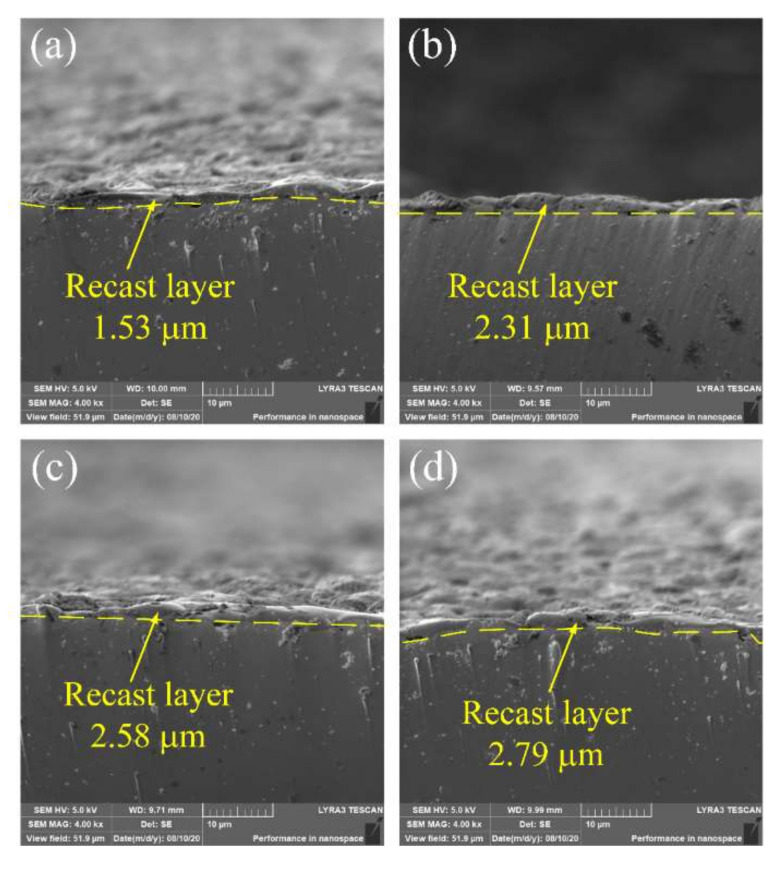
Cross-section images of EDM-treated TiNi SMA under pulse widths of (**a**) 1 μs, (**b**) 4 μs, (**c**) 7 μs, and (**d**) 10 μs.

The formation of the recast layer is affected by many factors, and the single pulse discharge energy is a key parameter for the formation of the recast layer [32]. Cross-section images of the recast layers in TiC-dielectric under different pulse widths are shown in Figure 11. As shown in Figure 11a–d, the thickness of the recast layer slightly increased with increasing pulse width. The discharge energy of micro-EDM was relatively low, which resulted in minimal changes to the thickness of the recast layer. The thickness of the recast layer measured in this experiment ranged from 1 to 3 µm, which was consistent with the research of Tan and Yeo [33]. The recast layer was composed of materials from the workpiece, electrode, and dielectric fluid. Increasing the pulse width caused more material to be melted and resolidified, thereby increasing the thickness of the recast layer.

**Figure 12:**
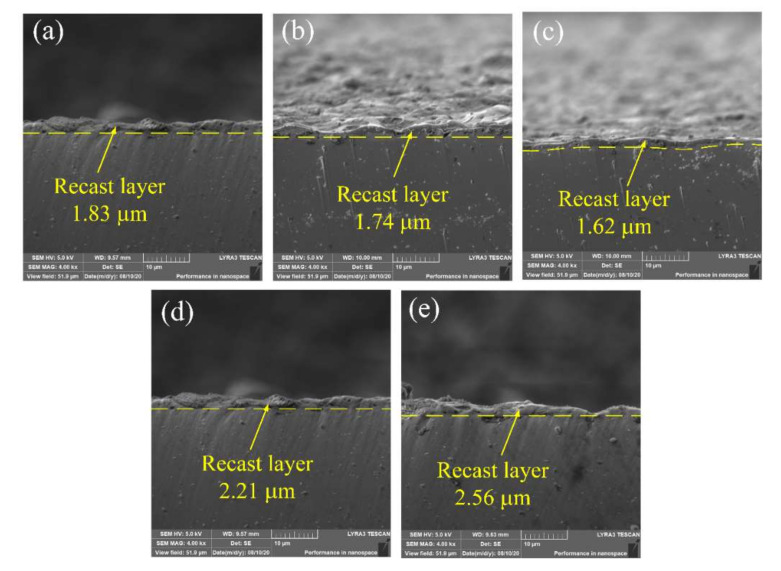
Cross section images of EDM-treated TiNi shape memory alloy (SMA) under the TiC concentrations of (**a**) 0 g/L, (**b**) 3 g/L, (**c**) 5 g/L, (**d**) 7 g/L, and (**e**) 10 g/L.

Figure 12a–e show cross-section images of the recast layers under the TiC concentrations of 0, 3, 5, 7, and 10 g/L, respectively. The thickness of the recast layer decreased with the addition of up to 5 g/L TiC to the dielectric and then increased with further increases in the TiC concentration. The MRR reached a maximum at 5 g/L TiC, and the melting material and deposited particles could be effectively removed. Therefore, a thin recast layer was formed in the 5 g/L TiC dielectric. The high concentration of TiC powder caused frequent secondary discharge and short circuits, which will generate a large amount of heat. This heat accumulation on the surface of TiNi SMA is beneficial for the formation of the recast layer.

### 4.6. Effect of Compound Composition on Microhardness

**Figure 13:**
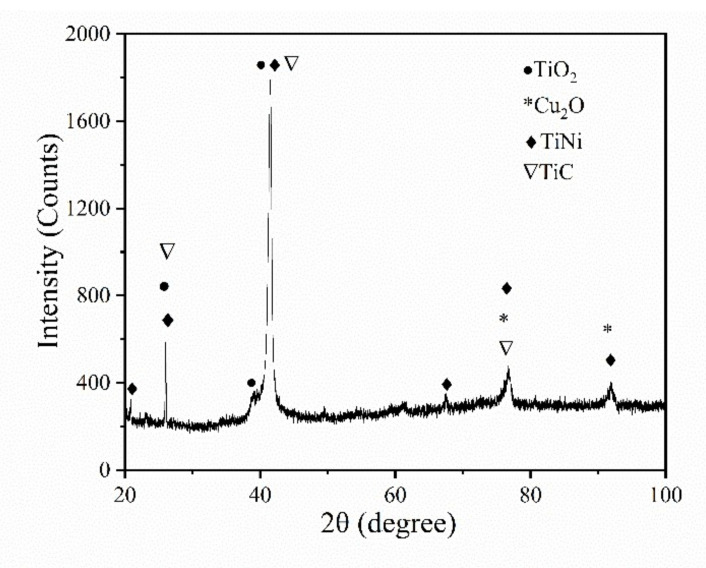
X-ray diffraction (XRD) pattern of TiNi SMA in 5 g/L TiC-dielectric.

Figure 13 shows the XRD pattern for the surface of the EDM-treated TiNi SMA with a pulse width of 4 µs, machining voltage of 80 V, and TiC powder concentration of 5 g/L. The results indicated that the EDM-treated surface layer consisted of TiO_2_, Cu_2_O, TiC, and TiNi phases. The presence of carbon and oxygen was associated with the decomposition of the TiC dielectric and the oxidation of molten metals. Cu electrode and TiC-dielectric medium were melted by EDM and deposited on the machined surface to form Cu_2_O and TiC. The most important observation in the XRD analysis was the formation of Ti_2_O. Jahan et al. [34] found that TiO_2_ has good biocompatibility and can provide a protective coating for biomedical implant applications. Hence, micro-EDM can be used to modify the surface of TiNi SMA and improve the biocompatibility of the titanium–nickel alloy.

**Figure 14:**
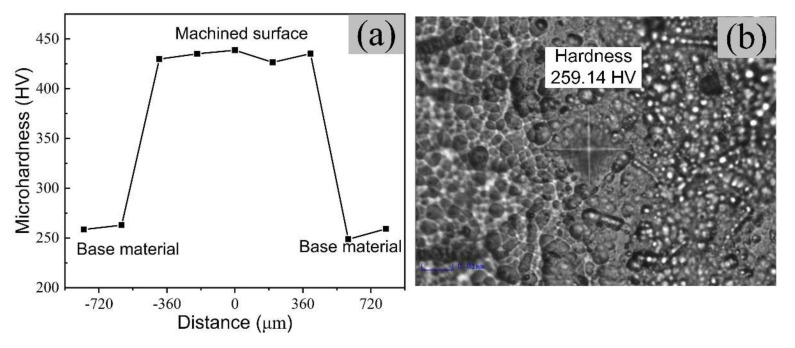
Measurement of surface microhardness of TiNi SMA: (**a**) microhardness curve of the surface and (**b**) microhardness measurement of the substrate surface.

The machined surfaces stand the continuous heating and cooling processes in EDM, which form a surface layer composed of the recast layer, heat affected zone, and base metal [34]. The recast layer has a great influence on the surface properties. Therefore, it is necessary to study the changes in surface microhardness. The microhardness curve at different distances from the center of the cavity and microhardness measurement of the substrate surface as shown in Figure 14a,b, respectively. The results show that the surface microhardness can reach 438.7 HV after micro-EDM, which is approximately 1.7 times the base material hardness. Chen et al. [35] recently showed that machined surfaces’ hardening effect originates from the recast layer. Combined with Figure 13, the XRD analysis revealed that the machined surface was composed of TiC, Ti2O, Cu2O, and TiNi, which could improve the surface’s microhardness.

## 5. Conclusions

The machining performance and feasibility of modifying the surface of a TiNi SMA through micro-EDM with the addition of TiC particles to the dielectric were discussed in this study. Discharge voltage waveforms demonstrated that the number of pulses in TiC-dielectric was significantly higher than in pure dielectric. The MRR, surface roughness, and thickness of the recast layer increased with an increase in discharge energy. MRR increased with an increase of TiC concentration, reaching a maximum at 5 g/L. Adding TiC particles to the dielectric can improve the surface finish by observing the surface morphology, and the machined surface in TiC-dielectric has smaller melting drops and craters compared to deionized water. The best surface finish occurred at a TiC concentration of 5 g/L. A layer ranging from 1 to 3 μm was obtained on the machined surface. The surface microhardness increased due to the formation of a recast layer containing TiC, Cu_2_O, and Ti_2_O; its hardness could reach 438.7 HV. Thus, this method can improve the wear resistance of the implant material, especially for orthodontic applications.

## Figures and Tables

**Table 1:** Experimental parameters.

Work Conditions	Description
Workpiece material	TiNi SMA
Electrode material	Brass (C2680)
Polarity	Positive
Dielectric fluid	Deionized water
Additive	TiC (2 μm)
Concentrations (g/L)	0, 3, 5, 7, 10
Duty (%)	50
Pulse durations (ms)	1, 4, 7, 10
Machining voltages (V)	60, 80, 100, 120
Cavity depth (mm)	100

**Table 2:** Elemental composition of the workpiece material.

Element	Ni	Ti	Nb	C	O	Other
Wt.%	50.9	48.9	0.025	0.036	0.043	<0.025

**Table 3:** Physical and mechanical properties of the TiNi shape memory alloy (SMA).

Workpiece Material	TiNi SMA
Density (kg/m^3^)	6450
Melting point (°C)	1310
Electrical resistivity (μΩ·m)	820
Modulus of elasticity (MPa)	42.3 × 103
Coefficient of thermal expansion (/°C)	11 × 10−6
Ultimate tensile strength (MPa)	880
Total elongation (%)	16

**Table 4:** Physical properties of TiC.

Property	Value
Density (kg/m^3^)	4930
Melting point (°C)	3140
Thermal conductivity (W/m·K)	21
